# Subtelomeric 5-enolpyruvylshikimate-3-phosphate synthase (EPSPS) copy number variation confers glyphosate resistance in *Eleusine indica*

**DOI:** 10.21203/rs.3.rs-2587355/v1

**Published:** 2023-02-22

**Authors:** Chun Zhang, Nicholas A. Johnson, Nathan Hall, Xingshan Tian, Qin Yu, Eric Patterson

**Affiliations:** 1 Guangdong Provincial Key Laboratory of High Technology for Plant Protection, Institute of Plant Protection, Guangdong Academy of Agricultural Sciences, Guangzhou, P.R. China; 2. Department of Plant, Soil, and Microbial Sciences, Michigan State University, East Lansing, MI, USA; 3. Australian Herbicide Resistance Initiative (AHRI), School of Agriculture and Environment, University of Western Australia (UWA), Perth, Australia

## Abstract

Genomic structural variation (SV) can have profound effects on an organism’s evolution, often serving as a novel source of genetic variation. Gene copy number variation (CNV), a specific form of SV, has repeatedly been associated with adaptive evolution in eukaryotes, especially to biotic and abiotic stresses. Resistance to the most widely used herbicide, glyphosate, has evolved through target-site CNV in many weedy plant species, including the economically important cosmopolitan grass, *Eleusine indica* (goosegrass); however, the origin and mechanisms of these resistance CNVs remain elusive in many weed species due to limited genetic and genomics resources. In order to study the target site CNV in goosegrass, we generated high-quality reference genomes for both glyphosate-susceptible and -resistant individuals, fine assembled the duplication of glyphosate’s target site gene enolpyruvylshikimate-3-phosphate synthase (EPSPS), and revealed a novel rearrangement of EPSPS into the subtelomeric region of the chromosomes, ultimately leading to herbicide resistance evolution. This discovery adds to the limited knowledge of the importance of subtelomeres as rearrangement hotspots and novel variation generators as well as provides an example of yet another unique pathway for the formation of CNVs in plants.

## 1. Introduction

*Eleusine indica* (Indian goosegrass) is one of the most economically important weed species in tropical and sub-tropical regions globally, commonly infesting cereals (especially rice), legumes, cotton, and vegetable crops, and it is also a common weed of turf systems. Decades of using the herbicide glyphosate for goosegrass control has applied enormous selective pressure for glyphosate resistance evolution. Glyphosate is a non-selective herbicide that targets the protein 5-enolpyruvylshikimate-3-phosphate synthase (EPSPS)^1^, an essential enzyme for aromatic amino acid synthesis in plants. In some cases, glyphosate resistance in goosegrass is caused by EPSPS target site mutations such as the Pro106 single mutations and the Thr102Ile and Pro106Ser (i.e. TIPS) double mutation^2^; however, increased EPSPS copy number variation (CNV)^3^, a type of genomic structural variation (SV), is the more frequent mechanism^3^ in this species. With both mechanisms present in goosegrass, some populations, or even individuals, can have both EPSPS CNV and target-site mutations^4^, with some, most, or all the copies of EPSPS having one or multiple mutations.

Genomic SV can have profound effects on an organism’s evolution^5^. As opposed to other forms of genetic variation, such as single nucleotide polymorphisms (SNPs) or insertions/deletions (InDels), SV does not always occur at a constant rate^6^. Instead, SV formation is punctuated and depends on several factors including the environment, transposable element activity, genetics, hybridization events, and the state of the epigenome. In plants, SV is a broad category and may include smaller-scale events like trans/cis-duplications, tandem duplications, and inversions as well as large scale events like chromosome arm inversions and even polyploidy^7^. SV that varies gene copy number (i.e., CNV) can have a direct impact on gene expression without changing the nucleotide sequence of the gene itself. Furthermore, additional gene copies often diverge over time and can eventually neo- or sub-functionalize, resulting in increased genetic diversity^8^.

Some regions of the genome, as well as some gene families, are especially prone to generating SVs and CNVs. Chief among these are regions of highly repetitive sequences where unequal crossing over happens frequently due to misalignment of sister chromatids, homologous chromosomes, and even non-homologous chromosomes. Chromosomes are often highly repetitive at their ends in the telomeres and subtelomeres. The subtelomeres are loosely defined and vary across taxa but are typically the next 50kb to 100kb of genome adjacent to the telomeres. Subtelomeres, while generally gene poor, can be sources of novel CNV events as crossing over can happen frequently. For instance, it has been previously shown in *Phaseolus vulgaris* that certain pathogen resistance genes exist in or near the subtelomere and that due to their proximity to the subtelomeres, these resistance genes are highly duplicated leading to certain pathogen resistance phenotypes^9^. This phenomenon is also relevant in monocotyledonous species. In allohexaploid bread wheat (*Triticum aestivum*), there is generally less synteny of genic regions among subgenomes in the subtelomeres compared to interstitial regions between the centromeres and subtelomeres, partially from high levels of CNV^10^. CNVs in subtelomeres are not limited to plants; in human subtelomeres, CNVs constitute around 80% of the most distal 100kb of the chromosomes^11^.

In plants, we are only now beginning to understand the importance of SV as a novel source of genetic variation due to the advent of ubiquitous and inexpensive genome sequencing technologies^12^. Projects like the 1001 Genomes Project^13^ are thoroughly investigating SV and its importance in *Arabidopsis thaliana*. Additionally, there have been other examples demonstrating the importance of SVs in the evolution of crops and non-crops and the massive effects that they can have on phenotypes^6,14–16^. One of the most striking examples of SV in action has been the evolution of glyphosate (Roundup) resistance in weedy species that effect row crop production. In cases where CNVs cause glyphosate resistance, the plant over-produces the EPSPS enzyme, to a degree that an enormous amount of glyphosate is needed to inhibit the entire EPSPS protein pool.

At least nine divergent, monocot and eudicot weed species have independently evolved glyphosate resistance via EPSPS CNV, an astounding example of convergent evolution^17^. Furthermore, each species has evolved these CNVs uniquely. The first EPSPS CNV was discovered in *Amaranthus palmeri* (palmer amaranth)^18^. It was eventually discovered that the EPSPS gene is being duplicated by a novel, extra-chromosomal, circular piece of DNA named “the replicon”^19,20^. The replicon independently replicates from palmer’s core genome and tethers itself to the core genome during cellular division^20,21^. Other weed species have duplicated EPSPS in more familiar ways; for example, EPSPS is duplicated in *Bassia scoparia* (kochia) in tandem and is thought to be the result of a combination of transposable element activity and unequal crossing over^22^. Efforts to identify mechanisms of CNV formation have been concentrated on dicot species in the Americas, although globally, weedy monocot species are more problematic.

Despite *E. indica*’s global economic importance, molecular biology and genomics research has remained difficult due to the lack of a quality reference genome and other molecular tools^23,24^. In this work, we generated a nearly end-to-end assembly of a glyphosate susceptible (GS) goosegrass individual and a near complete assembly of a glyphosate resistant (GR) individual. Furthermore, we use genomic resequencing, comparative genomics, and transcriptomics to identify the complete genomic region involved in goosegrasses EPSPS CNV event and provide insight in the mechanism driving increased copy numbers. This is the first in-depth investigation into the nature of EPSPS CNV in a grass species and is the first description of subtelomeric repeat driven herbicide resistance in any species.

## 2. Results and Discussion

### 2.1 Genome assembly, annotation, and overview

We assembled a chromosome-scale genome of a GS *E. indica* plant using PacBio HiFi and long-range interaction (Hi-C) datasets. The assembly consists of nine chromosomes spanning 509,878,742 base pairs estimated to be approximately 98.0% complete by Benchmarking Universal Single-Copy Orthologs (BUSCO)^25^ analysis. Gene models for this species were called using a *de novo* Isoseq dataset and predicted genes were prescribed function using homology and other protein domain predictive software. Ultimately 27,487 gene models were predicted in the GS *E. indica* genome. Additionally, we assembled another, near-chromosome level (>99% in 15 contigs), 541,164,105 base pair long genome using a separate PacBio HiFi dataset from a single GR individual, also estimated to be 98.0% complete by BUSCO. Using the same annotation pipeline, 29,090 genes were predicted in the GR *E. indica* genome. This resistant individual was confirmed to have increased EPSPS gene copy-number as its major glyphosate resistance mechanism in previous work via qPCR of EPSPS and EPSPS sanger sequencing^4^.

**Figure 1:**
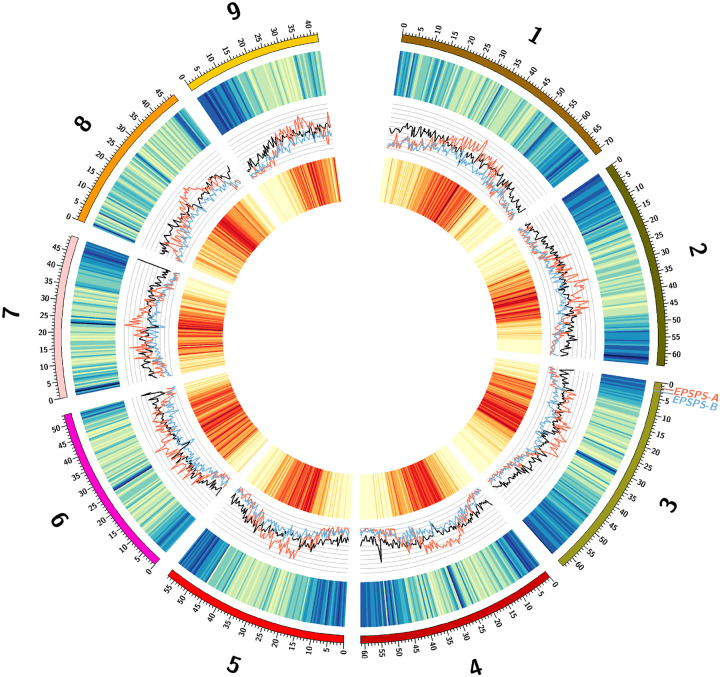
Overview of the glyphosate susceptible *Eleusine indica* genome. The Circos plot shows (a.) the length (Mb) of chromosomes one through nine as an index with corresponding (b.) gene-rich (blue) and gene-poor (yellow) genomic regions, (c.) Gypsy (red), Copia (blue), and other (black) transposable element family coverage across the genome (scale: 0–50%), (d.) transposable element rich (red) and transposable element poor (yellow) genomic regions, and the location of the EPSPS cassette (EPSPS-A & EPSPS-B).

On average, gene density and transposable element density vary inversely in both GR and GS genomes; gene density decreases near the centromeres but increases in the distal parts of the chromosome arms, with the opposite being true for transposable element density (Fig. 1). There are higher numbers of LTR transposons (i.e. Copia, Gypsy, and all other TEs), as well as other transposable elements, clustering at the centromeres and the most distal arms of each chromosome near the telomeres. The Gypsy superfamily is especially prevalent, having the highest transposable element density (Fig. 1). The GS genome contains 59.89% total repetitive sequence, including 2.53% DNA transposons and 40.21% retro-elements (38.29% of which are long terminal repeat elements). The GR genome contains 58.30% total repetitive sequence, including 2.08% DNA transposons and 35.71% retro-elements (33.91% of which are long terminal repeat elements). The most dominant transposon family in both genomes were classified as Gypsy transposons (~25%) in both genome assemblies, with Copia transposons being the second most abundant (~11%). All other transposons make up less than 2% of each genome. There were no large differences at the superficial scale in which we annotated repeat content between GS and GR.

**Figure 2:**
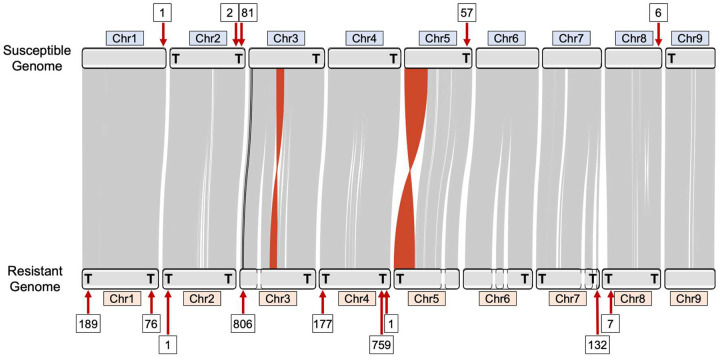
An ideogram of glyphosate susceptible and resistant genome alignment. Grey links indicate shared synteny between chromosome pairs. Red links indicate large inversions of synteny between the genomes. Black links represent Region-A and Region-B of the EPSPS-cassette in their native locations. Numbers in boxes above and below the ideogram indicate the number of copies of sub-telomeric repeats at each locus. A bold letter “**T**” on the karyotype represents ends of the chromosomes where the assembly reaches the telomeres.

*E. indica* has *Arabidopsis*-type telomere sequence (TTTAGGGn)^26^ that are tandemly repeated at the terminal ends of the chromosomes in up to 39,800bp long stretches. In the GS genome, chromosomes two, three, and four have these tandem telomeric repeats at one terminal end and the remaining chromosomes, one, five, six, seven, eight, and nine do not contain any terminal tandem telomeric repeats (Fig. 2). In the GR genome, chromosomes one, two, four, seven and eight begin and end with tandem telomeric repeats, while chromosomes three, five, six, and nine only have tandem telomeric repeats at one end of the chromosome (Fig. 2). This indicates we have captured most but not all the full-length chromosomes in the GR genome. The increase in telomere-to-telomere coverage in the GR genome compared to the GS may be explained by biological factors, such as increased homozygosity of the resistant line, or computational factors, such as different amounts of input PacBio or PacBio read N50 size. Most likely the highly repetitive chromosome ends and redundancy between the telomeric repeats makes further refinement difficult without the implementation of novel techniques specifically designed to resolve these regions.

The GS and GR assemblies are highly syntenic, as indicated by the linear arrangement of large (>10kb) lengths of nearly identical sequences that remain in order over entire chromosomes (Fig. 2). Due to the high amount of synteny between the assemblies, the slightly more fragmented GR genome has been manually ordered and named to maximize alignment with the GS genome. In the GR assembly, chromosomes three and five are composed of two contigs each while chromosomes six, and seven are comprised of three contigs, with the remaining chromosomes being in single contigs. Two large inversions were assembled in the GR genome at the ends of chromosomes five and six; and without Hi-C or optical mapping, we cannot say for certain how supported these inversions are or if they have any impact on the resistance phenotype.

**Figure 3:**
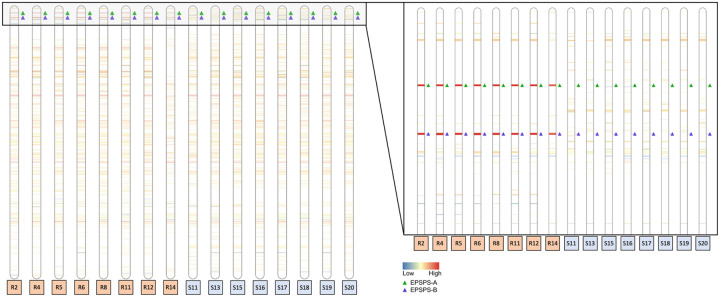
Copy number variation in chromosome three across eight GR and eight GS *Eleusine indica* individuals. The ideogram shows deletions below 0.25x of average read depth (blue color spectrum), copy number variation above 0.25 of average read depth and below 4x of average read depth and with a p-value greater than 0.01 (white), and duplications above 4x of average read depth (red color spectrum) across chromosome three in eight glyphosate-resistant (GR: R) versus eight glyphosate-susceptible (GS: S) *E. indica* individuals at a scale of (a.) full chromosome length (63,742,515 base pairs) and (b.) the first 5,000,000 base pairs of chromosome three. Band thickness is proportional to the length of the genomic region exhibiting copy number variation. The EPSPS cassette, duplicated consistently around 32x compared to average read depth in GR (R) individuals but is not duplicated in any of the GS (S) individuals, is marked by region: EPSPS-A with a green triangle and EPSPS-B with a purple triangle.

In both the GS and GR genomes, EPSPS, glyphosate’s target, was located approximately 1.6Mb from the telomere of chromosome three. In the GR genome, EPSPS was also identified in 23 short unscaffolded contigs, but always co-assembled in these un-scaffolded contigs with a copy of a sequence normally 2.4Mb from the telomere of chromosome three, about 1Mb downstream of EPSPS. We call the original location of EPSPS in the genome “Region-A,” and the region co-assembled with Region-A, “Region-B” for ease of discussion and clarity (Fig. 1; Fig. 3). Contrary to the tandem EPSPS duplications conferring glyphosate resistance in the weed *Bassia scoparia* (kochia)^22^, only one copy of Region-A was assembled at the native location on chromosome three in the GR genome, suggesting at least one initial trans-duplication event of the EPSPS must have occurred before subsequent duplications.

### 2.2 EPSPS structural variation

Eight GS and eight GR *E. indica* genomes were re-sequenced using PacBio HiFi to determine both the uniformity of the EPSPS CNV event in terms of structure but also in terms of other polymorphisms such as SNPs and InDels. This resequencing data was aligned to the GS genome and analyzed for 1) changes in read depth (indicating duplications or deletions) of certain regions that were uniform across the GR population and divergent from GS individuals, and 2) breaks in read alignment that describe rearrangements, translocations, inversions, and duplications. There were 34 shared predicted CNV duplication events among all GR individuals, 15 in the pseudomolecules and 19 in the unscaffolded contigs. CNV2 (Region-A; average read depth: 22.03) and CNV3 (Region-B; average read depth: 22.46) had nearly identical read depth in all individuals (Table 1; Fig. 3). Seven of the eight GR individuals had EPSPS read depths of approximately 25–29x above background except for sample R14 which only had 8x (Fig. 3). From this observation we believe this sample was a heterozygote, while the other seven GR individuals had copies in both the paternal and maternal genome. Additionally, these results indicate both that Region-A and Region-B are co-duplicated in every copy of the EPSPS CNV, and that Region-A and Region-B were translocated and fused prior to CNV proliferation. Four other CNV events (CNV21, CNV26, CNV 29, and CNV30) that were predicted to have high copy number (>20x) but are solely located in unscaffolded contigs that are mainly composed of highly repetitive DNA and/or transposons.

Region-A is approximately 35kb-long at the coordinates Chr3:1,666,751–1,701,750 in GS and Chr3:2,163,092–2,198,095 in GR. Region-A contains glyphosate’s target, EPSPS, as well as four other predicted genes: A390, A400, A410, and A440. Region-B is approximately 41kb-long at coordinates Chr3:2,719,751–2,760,750 in GS and Chr3:3,206,579–3,247,578 in GR with four predicted genes: B510, B520, B560, and B570 (Table 2). The TIPS double amino acid substitution of EPSPS was only found in sample R14. In R14, T102I was present in 12% of the 583 cDNA reads that mapped to EPSPS and P106S was present in 12% of 562 reads, each in approximately one out of the eight predicted copies. This indicates that as mentioned previously, this individual is heterozygous with one haplotype containing the EPSPS CNV and the other containing the TIPS mutation. Possibly associated with the TIPS mutation, a synonymous alanine substitution (GCA to GCG) was also found 28 amino acid residues upstream from T102I on all reads containing the TIPS mutations in the same 12% read coverage. The co-occurrence of TIPS and EPSPS CNV in this individual is not unexpected because it has been shown previously that *E. indica* individuals can have stacked resistance mechanisms and fitness penalties associated with mutations can be compensated for with unmutated copies of EPSPS. Such scenarios were predicted when the TIPS double mutation was first identified^2^ and later discovered in goosegrass^4^. Furthermore, it indicated that SNPs should also be carefully looked for in GR weeds with low EPSPS CNV frequency as a potential primary source of glyphosate resistance.

**Figure 4.**
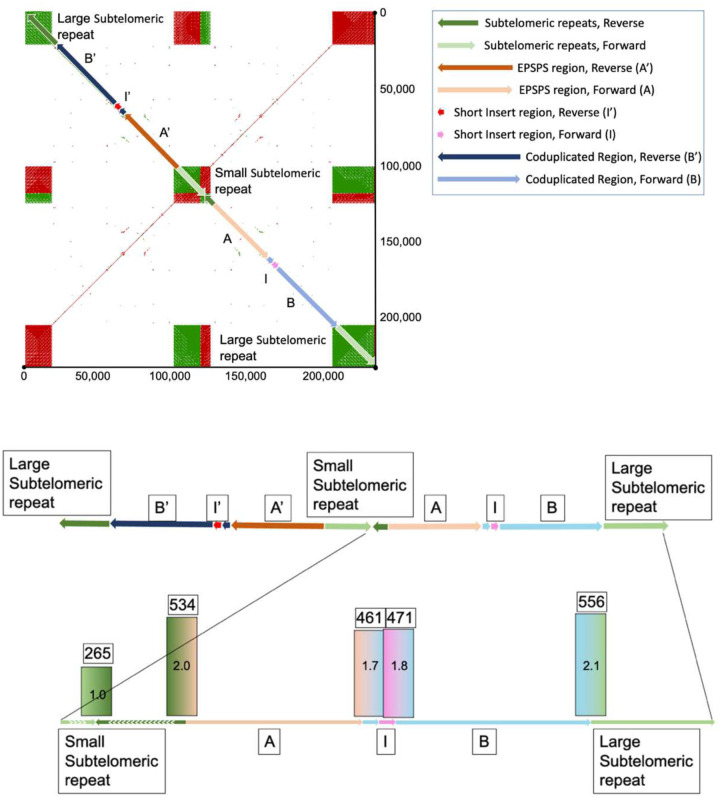
A self-alignment of the EPSPS-Cassette assembled from the GR genome. **A)** The cassette consists of several domains. The A-region is approximately 35kb and corresponds to Chr3:1666751–1701750 and contains the EPSPS gene itself. The B-region is approximately 41kb and corresponds to Chr3: 2719751–2760750. The I-region is a small, 450bp sequence inserted into the beginning of the B-region from an unknown origin. The entire cassette is assembled in reverse orientation, and it is denoted as A’, B’, and I’ in reverse orientation. A shorter stretch of subtelomeric repeats (472bp tandem repeat units) separates the forward and reverse copy of the cassette, and a larger stretch of repeats flanks the two cassettes on either end. **B)** PacBio reads from the resistant genome were aligned to the forward copy of the cassette to validate the junctions of each domain (STs-A, A-B, B-I, I-B, and B-ST) and to quantify their abundance. All junctions were confirmed to be present and assembled correctly. Furthermore, we confirmed that the inversion point of the cassette in the small subtelomeric repeat region was half as abundant (set at 1x coverage/265 reads). All other junctions were shown to be approximately twice as abundant (461–556 reads).

In GR individuals, Region-A and Region-B are fused and associated with a 3,396bp region of unknown origin inserted at the beginning of Region-B, labeled ‘Region-I’ (Figures 4a and 4b). Region-I contains no predicted genes or features of significance we can decern. Together, Region-A, Region-B, and Region-I makeup the entire ‘*EPSPS-Cassette*’, a structure not found in any of the glyphosate-susceptible individuals (Fig. 3a). Flanking the EPSPS-Cassette on both sides is a 452bp subtelomeric sequence that can also be found in the most distal part on various chromosomes in the susceptible and resistant *E. indica* genomes as well as to the subtelomere from other grass species. On one side of the cassette the subtelomeric sequence is repeated 12 times in the forward and 31 times in the reverse directions and serves to invert the EPSPS cassette. On the other side is a much larger array of repeats consisting of a minimum of 43 repeats in the forward and 294 repeats in the reverse directions (Fig. 3a). We were able to span and assemble across the small array of repeats; however, the larger array became ambiguous. Given previous fluorescent *in situ* hybridization (FISH) experiments EPSPS exists on two chromosomes in GR *E. indica*^27^. This information indicates that the EPSPS-Cassette is not scattered or widely dispersed but located in tandem on the ends of one or two chromosomes.

To verify the EPSPS-Cassette model presented here and specifically confirm each region-junction PacBio resequencing data were aligned to the EPSPS-Cassette (explicitly junctions checked are: Subtelomere-A, A-B, B-I, I-B, and B-Subtelomere). Around 545 PacBio reads were aligned to the manually assembled EPSPS-cassette model to support the Subtelomere-A and B-Subtelomere junctions, 466 reads support the A-B, B-I, and I-B junctions, and 265 reads support the subtelomere junction between the reverse and forward EPSPS-Cassettes (Fig. 3b). The number of reads supporting the inversion is almost exactly half of the number of reads that support all other junctions, indicating that the inversion exists half as much as the others. That is to say, the full-length cassette consists of one forward and one reverse copy of the A-B fusion, joined by the inverted, shorter subtelomeric repeat and flanked by much larger subtelomeric sequences on both sides.

**Figure 5:**
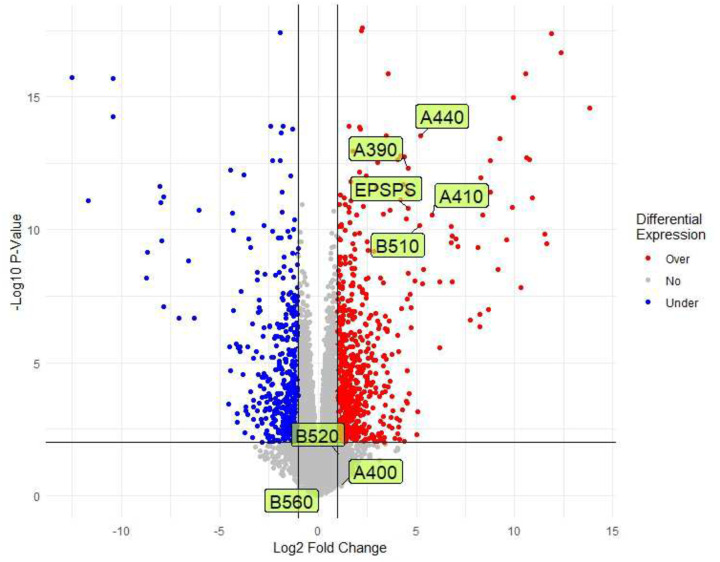
Differential expression of eight GR versus eight GS *Eleusine indica* individuals. The plot from RNA-Seq data shows over-expressed (red) and under-expressed (blue) genes in GR *E. indica* individuals with labels for all identified genes within the EPSPS cassette. Gene labels with a non-integer numerical value represent splice variants of the same gene. Genes below a *p*-value of 0.01 or a fold change value below two were considered not differentially expressed (grey) between the two treatment groups.

In addition, an RNA-seq experiment was performed to investigate gene expression changes driven by the EPSPS CNV. Although the entire EPSPS-Cassette and all genes within it are co-duplicated in GR individuals, four of the five genes in Region-A are significantly overexpressed (*p*-value<0.01 and fold-change>2), while only one out of four genes in Region-B is significantly overexpressed (Table 2, Fig. 5). Genes overexpressed other than EPSPS from Region-A include A410: A ribosomal subunit protein, A390: a tRNA-2’phosphotransferase, and A440: a protein of unknown function. Interestingly, homologs of A410 and A390 are also co-duplicated with EPSPS in *Bassia scoparia*, a eudicot weed with a tandemly duplicated EPSPS CNV^22^. Given the annotation of these proteins, it is unlikely they are directly involved in the EPSPS CNV formation. B510 is the only significantly overexpressed (log fold-change: 5.2, *p*-value: 6.9e-11) gene in Region-B (Fig. 7; Table 2). Gene B510 encodes a RadA-like protein, a type of protein known to be associated with EPSPS in glyphosate resistance in other prominent weed species such as kochia. This gene involvement in the formation of the EPSPS CNV is unknown; however, its overexpression indicates it is currently active. RadA proteins are DNA-dependent ATPase that process DNA recombination intermediates and are therefore involved in repairing DNA breaks^28^. These proteins are particularly interesting in the case of EPSPS duplication due to their role in catalyzing homologous recombination. Whether or not RadA is directly involved in the duplication of the EPSPS loci from various weed species or merely coincidental is open for examination.

### 2.3 Subtelomeres in *Eleusine indica*

Whole genome alignment reveals highly conserved subtelomeric repeat sequence from the EPSPS-Cassette near the ends of many of the assembled chromosomes in both the GS and GR goosegrass genomes (Fig. 2). Interestingly, over twice as many subtelomeres at higher average copy number in each region was assembled in the GR genome (Fig. 2). There is an especially high copy number of subtelomeric repeats in chromosomes one, three, four, and seven of the GR genome that could be sites of meiotic recombination and subtelomere rearrangement in and between chromosomes (Fig. 2)^29^. It has been previously shown in *Phaseolus vulgaris* that similar subtelomeric sequences are predisposed to unequal intra-strand homologous recombination^30^ commonly resulting in large duplications of whole pathogen resistance gene pathways.

**Figure 6:**
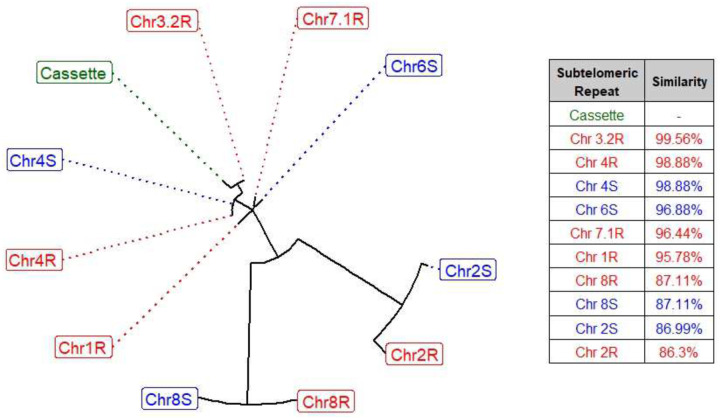
Relatedness of EPSPS-Cassette subtelomere sequence to chromosomal subtelomeric sequences of the GR and GS *Eleusine indica* genomes. The below plot shows the relatedness of subtelomeric sequences found on the glyphosate-resistant (GR: R, red) and glyposate-susceptible (GS: S, blue) *E. indica* genomes to the subtelomeric sequence found on the EPSPS-Cassette (green). Chromosomes at branch tips further from Cassette are less related to Cassette than chromsomes closer to Cassette. Branch distance is based on BLAST similarity. The sequence with the highest relatedness to the EPSPS-Cassette subtelomere sequence on each chromsome were used as representative sequences to make a tree.

The 452bp-long subtelomeric repeat unit that flanks the EPSPS-Cassette is most like the subtelomeric repeat region of second contig that comprises chromosome three from the GR genome (99.556%), indicating chromosome three is likely the location of the EPSPS-Cassette in GR plants. EPSPS is natively on chromosome three in both assembled genomes. The subtelomeric sequence of chromosome four in both the GS and GR genomes are also highly similar to the EPSPS-Cassette subtelomeric repeat region (98.884%). The subtelomeric repeats found on chromosomes one and seven of the GR genome and chromosome six of the GS genome are 95.778%−96.882% similar to the subtelomeric region of the EPSPS-Cassette, while chromosomes eight and two of both the GS and GR genomes are the least related (86.301%−87.113%). The subtelomeric repeats found on chromosomes four and eight of both genomes are identical (Fig. 6). Work by other researchers using FISH cytometry have shown that the EPSPS CNVs in goosegrass on one or possibly two chromosomes^27^. Given the sequence similarity of the subtelomeric repeat in the EPSPS-Cassette and the subtelomeres on chromosomes three and four, translocation of the EPSPS-Cassette between these two regions through non-homologous recombination seems feasible; however, we assembled chromosome four of the GR genome from telomere to telomere, completely through the subtelomeric region, and found no evidence of the EPSPS-Cassette on chromosome four in this population.

### 2.4 Subtelomeres in plant evolution

Subtelomeres of eukaryotic organisms are hotspots for adaptive evolution due to frequent, error-prone recombination events during meiosis that lead to rapidly changing genes. In common bean (*Phaseolus vulgaris*), segmental duplications, sometimes up to 100kb-long, of disease resistance genes located in the subtelomeres are facilitated by non-homologous end joining and frequent interchromosomal recombination^30^. The large duplications of these distal disease resistance genes allow for divergence of homologous genes into paralogs to allow novel resistance that accounts for the rapid evolution of pathogens. Analogous to the generation of novel disease resistance genes in the subtelomeres of common bean, CNV increases variation and diversity of virulence genes in the subtelomeres of several eukaryotic pathogens including *Plasmodium falciparum*, *Trypanosoma brucei* and *cruzi*, and *Pneumocystis carinii* and sugar metabolism genes in yeast^31^. Similarly, the translocation of genomic regions associated with herbicide or other abiotic stress resistance, like the EPSPS-Cassette to the subtelomeric region, may allow for novel abiotic resistance to develop from the rapid accumulation of variation between duplicate loci in addition to an increase in transcript abundance.

Duplications of the EPSPS-Cassette in the subtelomeres, especially in a grass species such as goosegrass, may not be unusual given subtelomeres propensity for generating and selectively maintaining novel variation in other grasses. In *Oryza sativa* (rice) the subtelomeres are theorized to be associated with high rates of transcription, recombination, and novel gene generation because they contain a large amount of highly similar paralogs, including some stress-response genes^32^. Despite the apparent stochastic nature of some rice subtelomeric regions, some subtelomeric regions in *Sorghum haplensis* (sorghum), *Brachypodium distachyon* (purple false brome), and several species of the *Oryza* genus exhibit duplications of the same genomic regions and preferential gene conversions within these duplicated regions, suggesting continuous concerted evolution and selective conservation of certain genes in subtelomeric regions^33^. Such precise selectivity is also evident in several *Avena* species (oats), where a subtelomeric, 12-gene cluster developed since the evolutionary divergence of *Aveneae* is maintained with gene order colinear to the biosynthetic pathway^34^. Given the importance of subtelomeres in other monocots for generating novel genetic variation and our findings, we have strong evidence that *E. Indica* EPSPS copy number variation is due to unequal crossing over in the subtelomeres of this species after an initial translocation event.

## 3. Conclusion

The large population size, plastic genomes, and large selection pressures exerted on weed species makes weeds an excellent system to study adaptive genome evolution. As we explore these genomes, we continuously find new ways that plant genomes innovate and overcome various stresses and discover exactly what makes weedy species like goosegrass such a successful survivor. The goals of this research were to both obtain high quality genomic resources for *E. indica*, a major, global weed, and to use those resources to investigate the genomic rearrangements and mechanism(s) that perpetrated EPSPS gene duplication and therefore glyphosate resistance in this species. By assembling both a GS and GR *E. indica* genome, genomic resequencing eight individuals of both populations, performing RNA-seq on eight individuals from each population, and manually curating the assembly surrounding the EPSPS locus, we have discovered that the EPSPS gene in GR *E. indica* has been duplicated, fused with another part of the genome, and inserted in one or more of the subtelomeric regions of the genome. We hypothesize that after this initial translocation and fusion, EPSPS duplication has carried on through unequal crossing over of the subtelomeres, facilitated by the high amounts of similarity between the subtelomeric sequence on the ends of chromosomes three. This is the first report of an herbicide resistance trait brought about by this type of genomic rearrangement and adds to both our knowledge of herbicide resistance evolution, and to the relatively limited information we have about the importance of subtelomeres as rearrangement hotspots and novel variation generators.

## 4. Methods

### 4.1 *Eleusine indica* tissue generation

Glyphosate-susceptible (GS) and glyphosate-resistant (GR) populations of *E. indica* that have been characterized in previous studies collected from Guangdong Province, China^4^. These populations were purified and self-pollinated for increased homozygosity and consistency of glyphosate susceptibility or resistance phenotypes. The GR population was confirmed to have EPSPS copy number variation by DNA quantitative PCR before purification.

Seeds of purified GS and GR biotypes were sown on wet filter paper in Petri dishes in a climate chamber at 28–30 °C, with 12h/12h light/dark period and 70% relative humidity. The two-leaf stage seedlings were transplanted into 28 × 54 cm trays (50 plants per tray) filled with potting soil and grown in a glasshouse. At the tillering stage, about ten individuals were randomly selected each from the GS and GR *E. indica* population and characterized. Three tillers of each plant were separated and repotted (one tiller per pot, 60 pots in total). One tiller of each plant was used for glyphosate resistance and susceptibility phenotyping, one for EPSPS CNV estimation and one for subsequent sequencing.

For glyphosate resistance and susceptibility phenotyping, one regrowth tiller (three days after tiller cloning) was treated with commercial glyphosate (41% glyphosate isopropylamine salt, 400 g ai ha-1 for GS and 1600 g ai ha-1 for GR), and GR (i.e., survivors) and GS (i.e., killed) phenotypes were determined three weeks after treatment. EPSPS CNV was again assessed in the resistant population to ensure the CNV event was still present before genomics work began. Leaf material from untreated tillers of corresponding resistant and susceptible plants was used for genomic DNA isolation using the Plant Genomic DNA kit (Trans Gen Biotech Beijing Co., LTD). Quantitative PCR was performed using published primer pairs and methods where EPSPS copy number was compared to the single copy acetolactate synthase (ALS) gene as the internal reference. The untreated tiller of a confirmed GS plant was used for genomic sequencing performed at Shanghai OE Biotech Co., Ltd (Shanghai, China).

### 4.2 Susceptible genome assembly.

The initial GS genome was generated from 112.2 Gb (223x) of raw PacBio Sequel II sequencing data and assembled using Falcon^35^. The resulting initial genome was 518,672,752 base pairs long in 239 contigs with a contig N50 of 18.8 Mb. Error corrections were conducted using the Arrow^36^ algorithm and a hi-coverage Illumina dataset. The error corrected assembly was compared to the NCBI^37^ nucleotide database using BLAST to identify possible bacterial or mammalian contamination, of which none was found. After polishing and removing contamination from the assembly, the assembly was 519,224,895 base pairs long in 239 contigs with a contig N50 of 18.8 Mb.

To further increase the continuity of the assembly and fix any possible large-scale errors, Hi-C data was obtained. The Hi-C library utilized the DpnII restriction enzyme (GATC cut sites) to generate sufficient digestion of the fixed DNA^38^. The final library was sequenced with Illumina HiSeq resulting in 150 base pair long paired end reads. Fastp (version 0.20; Chen et al., 2018) was used to clean the Hi-C paired end reads of adaptors and redundant PCR reads and perform analysis on the cleaned reads. Linker sequences and reads with greater than or equal to 5 N (not AGCT) bases were also removed. Sliding window (window size of 4 base pairs) was performed to excise windows with an average base quality score below 20. Filtered reads less than 75 base pairs long or with an average base quality score below 15 were removed. The resulting clean Hi-C reads had a total yield (G) of 93.48, 641,488,988 read pairs, a Q20% of 97.41%, a Q30% of 92.69%, and a GC content of 44.28%.

Juicer^39^ was used with the default parameters of both bwa-mem^40^ and 3D-DNA^41^ to scaffold the contigs of the PacBio only assembly. Contigs were then clustered by contact point proximity and sorted to generate a Hi-C interaction matrix that was imported into juicebox^42^ for visualization and manual inspection. The resulting matrix presented no abnormalities and contigs were able to be clustered into 9 chromosome scale scaffolds and 154 much smaller scaffolds. Gaps of 500 Ns were added between each contig to link the chromosomes for filling later. The scaffolded assembly was 519,302,895 base pairs long with an N50 of 57.27 Mb.

Finally, PBjelly^43^ was used to gap filling the assembly by aligning the original PacBio sequencing data to the Hi-C assembled genome. The gap filled assembly was now 522,502,607 base pairs long with a scaffold N50 of 57.37 Mb and a contig N50 of 42.10 Mb. Arrow^36^ was then used for self-comparison and another round of error corrections. Last, second-generation sequencing data was used for two rounds of Pilon^44^ error correction, resulting in a final assembly that was 522,557,097 base pairs long with a scaffold N50 of 57.37 Mb, a contig N50 of 42.10 Mb, and 62 total gaps. The final assembly was benchmarked for gene content using BUSCO)^25^of 1375 single-copy genes from the embryonic plants database the 1348 (98.0%) were identified as either single or multi copy.

### 4.3 Resistant genome assembly

The initial GR genome was generated from 28Gb (~53x) of PacBio HiFi^45^ sequencing data, assembled using HiCanu^46^ with a predicted genome size of 492Mb. The resulting initial genome was 541,164,105 base pairs long in 2014 contigs with a contig N50 of 47.4 Mb. Per the instruction of HiCanu, post-assembly error corrections were not conducted to avoid introducing errors and dropping below the 99.99% accuracy rating. A BUSCO assessment rated the resulting assembly as 98.0% complete. Contigs were aligned to the glyphosate-susceptible genome to identify chromosomes.

### 4.4 Genome annotation

Assembled GS and GR *E. indica* genomes were both annotated using a custom genome annotation pipeline developed by the International Weed Genomics Consortium genome annotation pipeline, as described below (https://www.weedgenomics.org/). First, repeat regions were annotated using RepeatModeler^47^ (version 2.0.2) and then masked using RepeatMasker^48^ (version 4.1.2) and bedtools^49^ (version 2.30.0) as a measure of data reduction before further annotation. Isoseq reads were then mapped to both repeat-masked genomes using Minimap2^50^ (version 2.24) to determine sites of transcription. The resulting Sequence Alignment Map (SAM) files were converted into Binary Alignment Map (BAM) files using the SAMtools^51^ (version 1.11) view command before being collapsed using cDNA Cupcake^52^ (version 28.0). The genomes, collapsed cDNA Cupcake outputs, repeat libraries from RepeatModeler, and a protein FASTA file from a close relative, *Eleusine corocana* (Phytozome genome ID: 560), were fed into MAKER^53^ (version) to predict the genomic coordinates of putative gene models. Genes that produced proteins under 32 amino acids long were removed from further annotation with only the longest proteins from each gene and unique untranslated regions (UTRs) used for functional annotation.

Functional annotation began by first selecting the longest isoforms from each gene using AGAT^54^ (version 0.8.0) and gffread^55^ (version 0.12.7). Longest isoforms sequence similarity searches were conducted using MMseqs2^56^ (version 4.1) with NCBI, UniRef 50^57^, and the InterPro^58^ database using InterProScan 5^59^ (version 5.47–82.0) locally. Protein localization was predicted using MultiLoc2^60^ (version 1.0). Using this pipeline, 27,487 genes in GS (BUSCO: 90.5%) and 29,090 genes in GR (BUSCO: 90.4%) were predicted.

### 4.5 Genome-resequencing and transcriptomics

Illumina 150bp paired-end sequences were generated from the DNA of eight GS and eight GR individuals from the above-named populations. DNA was extracted using leaf material from untreated tillers of corresponding GS and GR plants using the Plant Genomic DNA kit (TIANGEN, Beijing, China). DNA was sequenced using the Illumina HiSeq X Ten sequencing platform (Illumina Inc., San Diego, CA, USA) with an average of 30x coverage. Illumina reads were cleaned using FASTQ and aligned to the susceptible genome using HiSat2^61^ (version 2.1.0) using standard options for paired-end reads. CNVnator^62^ (version 0.4.1) was used to scan the read depth from the alignments in 5kb windows to roughly call regions of the genome that deviated significantly from the average read depth. CNVnator outputs were intersected using bedtools^49^ (version 2.30.0) intersect so that regions that were amplified in all eight GR individuals but none of the GS individuals were identified. Only two such regions were discovered. The region containing EPSPS on chromosome three was labeled “Region-A”, while the other, was labeled as “Region-B” for further analysis.

Illumina 150bp paired-end sequences were generated from the RNA of the same eight GS and eight GR individuals that were used for genome resequencing. RNA was extracted from the leave sheath material using the mirVana miRNA Isolation Kit (Ambion). Illumina reads were cleaned and aligned to the GS genome predicted transcriptome using HiSat2 (version 2.1.0) using standard options for paired-end reads. Alignments were converted into count tables using SAMtools (version 1.11). The count table was loaded into R (version 4.2.0) and differential gene expression was calculated using package edgeR^63^ (version 3.38.1) quasi-likelihood F-tests.

### 4.6 Investigation of the EPSPS-Cassette and subtelomeres

The EPSPS-Cassette model was resolved by first using BLAST to identify all contigs containing EPSPS, Region-A, and Region-B. Contigs of interest were self-aligned in YASS^64^ to visualize macrostructure, especially repeat structure. Contigs were manually assembled into putative models based on their repeat macrostructure. Contig junctions of the putative models were confirmed by aligning genomic reads to them using HiSat2 (version 2.1.0). The large subtelomeric repeat regions flanking the reverse-forward EPSPS-Cassette duplications were not able to be assembled completely using this method. The locations and relatedness of sequences similar to the 452bp subtelomeric repeat unit were found using Minimap2 (version 2.24) and BLAST.

### 4.7 Plot generation

Circos^65^ (version 0.69–9) was used to visually summarize the overall *E. indica* genome (Fig. 1). Coverage windows used to make the Circos tracks were generated using bedtools (version 2.30.0; Fig. 1). RIdeogram^66^ (version 0.2.2) was used to visualize duplications and deletions of EPSPS on chromosome three detected using CNVnator (version 0.4.1; Fig. 2). Synteny plots were made by aligning both genomes using MiniMap2 (version 2.24) and visualized using RIdeogram (version 0.2.2) in R (version 4.2.2; Fig. 3 & Fig. 5). The EPSPS Cassette was visualized using YASS (Fig. 4a & Fig. 4b). Differential expression between eight GS and eight GR *E. indica* individuals was visualized using ggplot2^67^ (version 3.4.0) in R (version 3.6.0). The subtelomere relatedness tree was generated using RAXML-NG^68^ (version 1.1.0), ggtree^69^ (version 3.6.2), cowplot^70^ (version 1.1.1), and ggplot2 (version 3.4.0) in R (version 4.2.2; Fig. 7).

## Figures and Tables

**Table 1:** Table of all duplicated CNVs shared in glyphosate resistant (GR) individuals that do not appear in any glyphosate-susceptible (GS) individuals.

CNV Event Number	Chromosome or Scaffold	Start	Stop	Length	Average Read Depth in GR
CNV1	Chrl	54,792,251	54,809,000	16,749	2.39
CNV2 (A)	Chr3	1,666,751	1,701,750	34,999	22.03
CNV3 (B)	Chr3	2,719,751	2,767,250	47,499	22.46
CNV4	Chr4	30,001,501	30,024,250	22,749	3.31
CNV5	Chr4	32,703,001	32,731,250	28,249	9.19
CNV6	Chr5	10,538,001	10,552,000	13,999	2.23
CNV7	Chr5	13,291,251	13,298,500	7,249	2.22
CNV8	Chr6	120,001	124,750	4,749	2.18
CNV9	Chr6	142,751	153,250	10,499	3.40
CNV10	Chr7	27,384,251	27,401,750	17,499	2.03
CNV11	Chr7	35,146,001	35,150,750	4,749	2.14
CNV12	Chr7	40,076,251	40,112,750	36,499	3.68
CNV13	Chr8	42,437,251	42,510,250	72,999	2.06
CNV14	Chr9	11,015,251	11,024,750	9,499	5.86
CNV15	Chr9	18,044,251	18,072,250	27,999	2.80
CNV16	Scaffold12	46,751	94,500	47,749	3.29
CNV17	Scaffold12	121,751	135,250	13,499	4.66
CNV18	Scaffold26	99,251	118,500	19,249	4.71
CNV19	Scaffold29	32,251	58,000	25,749	3.04
CNV20	Scaffold29	88,251	124,750	36,499	4.28
CNV21	Scaffold30	13,251	123,250	109,999	32.46
CNV22	Scaffold35	10,001	120,500	110,499	2.93
CNV23	Scaffold36	1	38,500	38,499	3.04
CNV24	Scaffold36	52,001	88,750	36,749	2.16
CNV25	Scaffold36	104,751	117,250	12,499	5.38
CNV26	Scaffold44	1	93,250	93,249	47.34
CNV27	Scaffold45	1	50,250	50,249	2.07
CNV28	Scaffold45	68,001	91,000	22,999	5.88
CNV29	Scaffold47	1,251	80,000	78,749	45.20
CNV30	Scaffold49	1	77,750	77,749	65.48
CNV31	Scaffold51	1	23,250	23,249	2.25
CNV32	Scaffold55	1	16,250	16,249	3.84
CNV33	Scaffold56	37,251	65,500	28,249	6.15
CNV34	Scaffold58	48,501	64,000	15,499	4.42

**Table 2:** RNA-Seq data of genes contained within the EPSPS-Cassette

Gene ID	Label	Annotation	Coordinates		RNA-Seq	
Glyphosate susceptible Glyphosate resistant			Start GS Start GR	Stop GS Stop GR	logFC	P-value
Region A						
EleInSChr3g081370 EleInRChr3_2g092340	EPSPS	EPSPS	1,669,076 2,165,535	1,673,225 2,169,359	4.6	1.6e-11
EleInSChr3g081410 EleInRChr3_2g092360	A410	Ribosomal subunit protein	1,673,439 2,169,409	1,675,161 2,171,640	5.8	2.8e-11
EleInSChr3g081390 EleInRChr3_2g092350	A390	tRNA 2’-phosphotransferase 1	1,675,833 2,172,099	1,680,441 2,176,989	4.6	4.9e-13
EleInSChr3g081400** None.	A400	Unknown protein	1,682,414 2,178,757	1,687,866 2,184,208	1.2	0.42
EleInSChr3g081440 EleInRChr3_2g080580	A440	Unknown protein of *E. coracana*	1,695,565 2,192,031	1,701,288 2,198,135	5.2	3e-14
Region B						
EleInSChr3g082510 EleInRChr3_2g221600	B510	DNA repair protein RadA-like	2,724,808 3,211,487	2,735,415 3,224,071	5.2	6.9e-11
EleInSChr3g082560 EleInRChr3_2g 119210	B560	6-phosphofructokinase 1 (PFK) gene, complete CDS	2,742,394 3,229,211	2,747,873 3,234,785	0.41	0.78
EleInSChr3g082520** Not Annotated in R	B520	Putative dual specificity protein phosphatase DSP8	2,748,924 3,235,752	2,752,196 3,239,024	1.1	0.03
EleInSChr3g082570* EleInRChr3_2g091630	B570	E3 ubiquitin-protein ligase 1	2,758,205 3,245,033	2,759,136 3,245,964	-	-

* Filtered from differential expression plot during edgeR processing

** Not annotated in GR genome. Coordinates from BLAST of GS gene against GR genome.

## Data Availability

The assembled genomes, associated GFF annotation files, and all functional annotation information are publicly available through the International Weed Genomics Consortium online database, ‘Weedpedia’ (https://weedpedia.weedgenomics.org/). Raw sequencing data has been submitted to the National Center for Biotechnology Information (NCBI) Sequence Read Archive (SRA); Genome resequencing data with the accession numbers SRR23364316-SRR23364331 and RNA-seq data with the accession numbers SRR23372273-SRR23372288.
